# Flow versus resistance: hemodynamic phenotyping of pulmonary hypertension in infants with shunts and concurrent lung disease

**DOI:** 10.3389/fped.2026.1880632

**Published:** 2026-09-14

**Authors:** Tania Gonzalez, Charles T. Simpkin, Paula Dias Maia, Jamie L. Archambault, Anne Taylor, D. Dunbar Ivy, Steven H. Abman, Benjamin S. Frank

**Affiliations:** 1 University of Colorado School of Medicine, Aurora, CO, United States; 2 Department of Pediatrics, Section of Cardiology, University of Colorado School of Medicine, Aurora, CO, United States; 3 Department of Pediatrics, Children’s Healthcare of Atlanta/Emory University, Atlanta, GA, United States; 4 Department of Pediatrics, Section of Neonatology, University of Colorado School of Medicine, Aurora, CO, United States; 5 Pediatric Heart Lung Center, Department of Pediatrics, University of Colorado, School of Medicine, Aurora, CO, United States

**Keywords:** bronchopulmonary dysplasia, congenital heart disease, echocardiography, neonatal hemodynamics, pulmonary hypertension

## Abstract

Neonatal pulmonary hypertension (PH) can be categorized into three distinct physiologies: resistance-driven precapillary pulmonary arterial hypertension (PAH), flow-driven precapillary PH, and postcapillary PH. We present eight patients, divided into three groups: premature infants with BPD, infants with chronic respiratory failure secondary to CDH or genetic syndrome, and an infant with a multilevel shunt. In each case, echocardiography was an important, non-invasive tool for screening risk of PAH. However, many of the common echocardiographic parameters used to assess PAH are confounded by volume and pressure loading due to cardiac shunts. These factors may lead to inaccurate assessments of patient hemodynamics and inappropriate treatment decisions. This series highlights challenging clinical cases of at-risk neonates wherein echocardiographic data was initially suggestive of PAH physiology, but subsequent invasive hemodynamic testing revealed significant flow from cardiac shunts, specifically atrial septal defects, ventricular septal defects, and/or patent ductus arteriosus, were main contributors to their clinical presentation.

## Introduction

Pulmonary hypertension (PH) occurs in 14%–25% of premature neonates and is especially prevalent among infants with developmental lung diseases (1–4). For example, severe bronchopulmonary dysplasia (BPD) is associated with a reported prevalence as high as 58% (1–3, 5).

Neonatal PH can be categorized into three distinct physiologies: resistance-driven precapillary pulmonary arterial hypertension (PAH, due primarily to increased pulmonary vascular resistance), flow-driven precapillary PH (due primarily to increased pulmonary blood flow), and postcapillary PH (increased left atrial pressure or pulmonary capillary wedge pressure leading to elevated pulmonary arterial pressures) (6–8). The first phenotype is seen in neonates with developmental lung disease where lung hypoplasia coupled with impaired vascular development contribute to vascular remodeling and ultimately elevated pulmonary vascular resistance (PVR) (3, 9, 10). The second phenotype can occur in the context of both pre-tricuspid (atrial septal defects, ASD) and post-tricuspid (patent ductus arteriosus, PDA, and ventricular septal defect, VSD) shunts. Although prolonged exposure to intracardiac shunts can eventually result in pulmonary vascular remodeling and PAH, in early infancy the predominant effect of excess pulmonary blood flow is elevated pressure within the pulmonary vascular bed, without a corresponding increase in PVR (8). Post capillary PH in neonates, whether due to pulmonary vein disease or left heart disease, presents a significant diagnostic and therapeutic challenge as there is often mixed physiology of pulmonary vascular disease and fixed obstruction to pulmonary venous return (11).

Echocardiographic markers of PH, including peak tricuspid regurgitation jet velocity (TR-Vmax) and systolic septal flattening, are fundamentally surrogate measures of right-sided pressure loading. In pediatric patients with PH without intracardiac shunts, however, these parameters have been shown to correlate with invasively measured pulmonary vascular resistance (PVR) and disease severity (12–14). Consequently, echocardiographic evidence of elevated right ventricular pressure is often interpreted as evidence of increased pulmonary vascular resistance. This assumption is frequently extrapolated to infants with suspected PH from other etiologies. Particularly in infants where both developmental lung disease and cardiac shunts are present, distinguishing the relative contributions of each condition to the overall hemodynamic profile is challenging. Such cases underscore a key limitation of echocardiography—wherein commonly utilized parameters provide imprecise estimates of right sided pressures, and do not directly quantify PVR. This is clinically important, as accurately distinguishing infants with large shunts that elevate pulmonary pressures despite normal or near-normal PVR from those with more significant elevations in PVR can greatly alter patient management. While the latter state often benefits from pulmonary vasodilator therapy, the former are at risk for pulmonary over-circulation with these same interventions and may even benefit from shunt closure. Invasive hemodynamic assessment via cardiac catheterization remains the gold standard for differentiating these two pathophysiologic states (15, 16). However, this approach is significantly limited in this population due to risks associated with the patient size, clinical instability, need for anesthesia, and the procedure itself (17–19).

In this report, we present several challenging cases in which initial echocardiographic findings suggested the presence of PH and raised concern among members of the treating team that increased pulmonary vascular resistance may be driving their physiology, but subsequent cardiac catheterization revealed a hemodynamically significant cardiac shunt as a substantial contributor to the PH. We aim to highlight the diagnostic complexities, need for multi-modality evaluation, and management challenges in this high-risk infant population.

## Methods

Cases were retrospectively identified as patients 1) aged 0 to 5 years at the time of initial suspicion for pulmonary hypertension, 2) having a diagnosis of congenital heart disease including simple intracardiac or vascular shunts (e.g., ASD, VSD, PDA), and were 3) initially evaluated for PH using echocardiography with subsequent cardiac catheterization to further investigate the hemodynamics at Children's Hospital Colorado between January 1, 2020 to November 30, 2025. Decisions regarding pulmonary vasodilator therapy, supplemental oxygen, inhaled nitric oxide, and other supportive measures were made on an individual basis by the medical teams according to clinical judgment and the patient's condition at the time of care. We purposefully selected cases where, in retrospect, the clinical team's assessment of patient physiology based on non-invasive echocardiographic evaluation differed from that based on subsequent cardiac catheterization. While this inherently creates a biased sample, the goal was to highlight the clinical reality wherein the bedside assessment of a patient's physiology based on echocardiography may be incorrect in the presence of a large shunt.

Echocardiographic data from within one month prior to catheter studies and 2–6 months post-intervention were reviewed and tabulated. Selected markers of PH including right ventricular size, function, Doppler estimates of pulmonary pressures and septal flattening were recorded. Hemodynamic data from the diagnostic catheterization assessment prior to resolution of shunt physiology was recorded for each subject. PH was defined as mPAP >20 mmHg as described by the World Symposium on Pulmonary Hypertension (20). This project was approved by the Colorado Multiple Institution Review Board (COMIRB), under Protocol #22-1476 with a waiver of consent as it was conducted retrospectively.

## Results

### Patient characteristics

During the study period, we identified eight infants who met inclusion criteria. Each subject was initially identified as having signs of PH by echocardiography despite vasodilator therapy and subsequently underwent invasive hemodynamic testing that revealed hemodynamically significant shunts with higher Qp and lower PVR than anticipated. Commonly identified echo markers of PH were TR-Vmax, systolic septal flattening, low velocity (or bidirectional PDA or VSD shunts). No patient had echocardiographic measurements of Qp:Qs performed prior to catheterization. These eight patients were categorized into three groups: 1) four former preterm infants with BPD; 2) three term infants with respiratory failure secondary to congenital diaphragmatic hernia (CDH) or a genetic syndrome; 3) a single term infant with acute respiratory failure due to viral illness. Full patient demographics and clinical course are found in Table 1, while selected echocardiographic and hemodynamic findings for each patient are presented in Table 2. Baseline respiratory support and baseline therapies discussed are defined as the respective support or therapy reported within 24 h prior to each patient's intervention, whether transcatheter procedure or surgery.

**Table 1:** Demographic and clinical information.

Group	Case	Sex	GA (wks)	Birth weight (kg)	Age/Wt at Cath (kg)	Underlying Diagnoses	Respiratory Diagnoses	Baseline Respiratory Support	Baseline Medical Therapies				Procedure	Change in Medical Therapy	Respiratory Support at Discharge
									PDE-5i	ERA	iNO	Diuretic			
Group 1	1	M	25	0.6	4mo/3.7	T21, ASD, PDA (previously ligated)	Grade 2 BPD	LFNC (day) CPAP (night)	X			X	ASD closure	None	LFNC (day), BiPAP (night)
	2	F	24	0.78	3mo/3.1	ASD, VSDs (AM), PDA	Grade 3 BPD	BiPAP				X	PDA closure	Sildenafil started	LFNC
	3	F	25	0.67	5mo/4.7	ASD, VSD (MM), PDA (previously ligated)	Grade 3 BPD, CRF	HHFNC				X	ASD closure	None	LFNC (day), CPAP (night)
	4	F	28	0.95	7mo/5.1	ASD, PDA, PVS (previously ballooned)	Grade 1 BPD	LFNC	X				PDA ligation	None	LFNC
Group 2	5	F	34	1.92	5mo/7.4	Heterotaxy Syndrome (left-isomerism), ASD, PDA (previously ligated)	Bronchomalacia, CRF	Trach/Vent			X		None	iNO stopped	Trach/Vent
	6	F	39	3.1	1mo/3.8	Kleefstra syndrome, ASD, VSD (AM), PDA	CRF	BiPAP	X		X	X	ASD closure, PDA ligation	iNO stopped	LFNC
	7	M	38	3.2	16mo/11.3	ASD, PAH (diagnosed by prior cath)	CDH, CRF	LFNC (day), BiPAP (night)	X	X		X	ASD closure	None	LFNC
Group 3	8	F	41	2.72	9mo/7.1	VSD (PC), ASD, unroofed CS	ARF (Viral)	LFNC	X			X	VSD closure Repair of unroofed CS	None	RA

GA, gestational age; wks, weeks; kg, kilograms; mo, months; PDE-5i, phosphodiesterase type 5 inhibitor; ERA, endothelin receptor antagonist; iNO, inhaled nitric oxide; ACEi, angiotensin-converting enzyme inhibitor; M, male; F, female; T21, Trisomy 21; ASD, secundum atrial septal defect; PDA, patent ductus arteriosus; VSD (PC), perimembranous central ventricular septal defect; AM, anterior trabecular muscular VSD; MM, midseptal muscular VSD; CS, coronary sinus; PVS, pulmonary vein stenosis; BPD, bronchopulmonary dysplasia; CRF, chronic respiratory failure; ARF, acute respiratory failure; CDH, congenital diaphragmatic hernia; LFNC, low flow nasal cannula; HHFNC, heated high flow nasal cannula; CPAP, continuous positive airway pressure; BiPAP, bi-level positive airway pressure; RA, room air. Baseline respiratory support and baseline therapies are defined as the respective support or therapy reported within 24 h of the described procedure or surgery.

**Table 2:** Abbreviated echocardiography and invasive hemodynamic data.

Group	Case	Pre-Intervention Echo Summary	Post-Intervention Echo Summary	Catheterization condition	mPAP (mmHg)	LAP or PCWP (mmHg)	PVRi (Wu*m^2^)	Qp:Qs	Interpretation and Outcome
Group 1	1	TR-Vmax 4.3 m/s; moderate septal flattening; large L→R ASD	TR-Vmax 1.9 m/s; ASD closed	25%FiO2	25	7	5.05	1.4	Reactive PH with increased Qp:Qs → ASD closure
				100%FiO2 + 40 ppm iNO	20	6	1.55	2.64	
	2	Mild septal flattening; L→R PDA, moderate L→R ASD, small L→R VSD	Mild septal flattening; PDA closed	30% FiO2	30	7	3.01	3	PDA closure improved Qp:Qs, permitted more aggressive PAH treatment
				s/p PDA closure	26	7	3.49	1.43	
	3	Moderate septal flattening; moderate L→R ASD	Mild septal flattening; ASD closed; RV improved	30% FiO2	25	–	2	1.79	ASD closure
	4	TR-Vmax 4.8 m/s; severe septal flattening; bidirectional PDA, small bidirectional ASD	Moderate septal flattening; PDA ligated	25% FiO2	30	9	3.60	1.67	PVS + PDA physiology → PDA closure
				post-angioplasty + 40% FiO2	27	6	3.53	1.89	
Group 2	5	TR-Vmax 4.7 m/s; moderate septal flattening; large bidirectional ASD	TR-Vmax 3.3 m/s; moderate septal flattening; L→R ASD	50%FiO2 + 20 ppm iNO	34	15	2.74	2.0	Complex CRF physiology → iNO discontinued
				50% FiO2	35	15	2.93	0.82	
	6	TR-Vmax 3.3 m/s; severe septal flattening; L→R PDA, large L→R ASD, small L→R VSD	TR-Vmax 3.0 m/s; mild septal flattening; ASD + PDA closed	30%FiO2 + 10 ppm iNO	43	6	6.4	1.1	Reactive PH with increased Qp:Qs → ASD + PDA closure
				70% FiO2 + 40 ppm iNO	28	5	1.2	3.3	
	7	TR-Vmax 3.7 m/s; mild septal flattening; moderate L→R ASD	TR-Vmax 2.9 m/s; no septal flattening; ASD closed	30% FiO2	24	7	3.3	1.24	ASD closure
				100% FiO2	25	–	2.12	1.7	
Group 3	8	TR-Vmax 4.0 m/s; moderate septal flattening, unroofed CS, moderate L→R VSD	TR-Vmax 2.9 m/s; mild septal flattening; VSD closed	25% FiO2	35	10	3	2.27	VSD closure, repair of unroofed coronary sinus

mPAP, mean pulmonary artery pressure; LAP, left atrial pressure; PCWP, pulmonary capillary wedge pressure; PVRi, pulmonary vascular resistance index; Qp:Qs, pulmonary-to-systemic flow ratio; TR-Vmax, tricuspid regurgitation maximal velocity; ASD, atrial septal defect; PDA, patent ductus arteriosus; VSD, ventricular septal defect; FiO2, fraction of inspired oxygen; iNO, inhaled nitric oxide; ppm, parts per million; PH, pulmonary hypertension; L→R, left-to-right shunt; PVS, pulmonary vein stenosis; CRF, chronic respiratory failure.

All cases required significant modification to their clinical management plan as a result of this invasive hemodynamic testing, ranging from changes to their medication regimen to attempting shunt closure. Seven patients successfully underwent shunt closure following hemodynamic testing—including ASD closures (six), PDA closure (three), and VSD closure (one). In the majority of patients, echocardiographic measures of PH improved after shunt intervention. All patients aside from one had isolated precapillary pulmonary hypertension by invasive hemodynamics. One patient was acutely weaned off inhaled nitric oxide (iNO) following catheterization. The majority of patients who were on enteral pulmonary vasodilator medications at the time of catheterization were maintained on this therapy at the time of discharge. Five of the seven patients who underwent shunt intervention had improved respiratory support by the time of discharge.

### Group 1: premature infants with BPD (cases #1-4)

This group consisted of four former preterm infants born at 24–28 weeks of gestation with a birth weight 600 g to 950 g, ranging from 3 to 7 months old at the time of catheterization. Two infants (Cases #1 and #3) each had left-to-right ASDs and a history of PDA that had previously been ligated at other institutions. Both demonstrated persistent moderate systolic septal flattening and evidence of right ventricular dilation by echo despite PDA closure and optimization of pulmonary status and were therefore taken to the catheterization lab. Case #1 was found to have mildly elevated mean pulmonary artery pressures (mPAP 25 mmHg) and indexed pulmonary vascular resistance (PVRi 5.05 Wu*m2) on baseline conditions, both of which improved significantly with vasoreactivity testing (the addition of oxygen and iNO) to a mPAP of 20 mmHg and PVRi of 1.55 Wu*m2. Results also showed the baseline ratio of pulmonary-to-systemic blood flow (Qp:Qs) was 1.4:1 which increased to 2.6:1 upon vasoreactivity testing. With this data, percutaneous closure was attempted but failed due to anatomic constraints and they were referred for planned surgical closure for ASD, which ultimately happened around 12 months of life. Similarly, Case #3 had a baseline mPAP of 25 mmHg, a normal PVRi of 2.00 Wu*m^2^ and a Qp:Qs of 1.8:1, prompting catheter-based closure of their ASD.

Cases #2 and #4 both had moderate sized PDAs. By echo, Case #2 had a low-velocity left-to-right shunt and Case #4 had a bidirectional shunt, suggesting systemic or supra-systemic pulmonary artery pressures, respectively. However, under general anesthesia Case #2 demonstrated modest elevations in both mPAP (30 mmHg) and PVRi (3.01 Wu*m2) with a significantly elevated Qp:Qs of 3.0:1. Based on this data the PDA was closed in the catheterization lab. Case #4 had similar elevations in mPAP and PVRi with a slightly lower baseline Qp:Qs (1.7:1) in the setting of concurrent pulmonary vein stenosis, which required balloon angioplasty. Ultimately, based on the size of the PDA and the presence of a 1.9:1 shunt after pulmonary vein angioplasty, this patient underwent successful surgical PDA ligation.

After shunt closure, the echocardiographic evidence of PH (particularly systolic septal flattening) improved for all patients in this group. Cases #1 and #4 did not show significant improvement in respiratory support whereas Case #2 was able to wean from chronic non-invasive positive pressure ventilation (NIPPV) to daytime low-flow nasal cannula. Case #3 was ultimately transitioned from chronic heated high flow (HHFNC) to daytime low-flow nasal cannula and nocturnal NIPPV which facilitated their discharge. All four patients were ultimately discharged home and there were no reported morbidities associated with their cases.

### Group 2: infants with chronic respiratory failure secondary to CDH or genetic syndrome (cases #5-7)

Infants in this group were born between 34 and 39 weeks at 1,920 g and 3,200 g, with their catheterization studies between 1 and 16 months of age. Case #5 had heterotaxy syndrome, a large, bidirectional ASD, and a previously ligated PDA. There was high velocity TR Vmax (4.7 m/s), moderate septal flattening, and moderate right ventricular (RV) dilation on echocardiography despite ligation of the PDA and treatment with iNO, prompting catheterization studies. In the baseline condition (50% FiO2, 20 ppm iNO), mPAP was elevated to 34 mmHg, PVRi was nearly normal (2.74 Wu*m2), and Qp:Qs was elevated at 2:1. Following intra-catheterization wean of iNO, mPAP and PVRi were essentially unchanged while the Qp:Qs fell to 0.8:1 and the pulmonary capillary wedge pressure was elevated at 15 mmHg, suggesting a component of post-capillary PH. During this cardiac catheterization ASD closure was attempted but ultimately aborted due to unfavorable anatomy. Surgical ASD closure was considered but ultimately deferred.

By echocardiography, Case #6 had severe septal flattening and moderate RV dilation in the setting of a small, low-velocity PDA, large ASD, and a small VSD all with left-to-right flow, despite treatment with multiple vasodilators including iNO. This patient carried a diagnosis of Kleefstra syndrome and required chronic NIPPV. Catheterization demonstrated moderate PAH with right ventricular systolic pressure around 77% systemic, an elevated mPAP at 43 mmHg and PVRi (6.4 Wu*m2) on 30% FiO2 and 10 ppm of iNO. After administration of 40 ppm of iNO, mPAP and PVRi significantly improved to 28 mmHg and 1.2 Wu*m2, with an increase in the Qp:Qs from 1.1:1 to 3.3:1. Thus, the patient was referred for surgical PDA ligation and ASD closure.

Case #7 was a 9-month-old with a history of repaired CDH and chronic respiratory failure requiring nocturnal NIPPV. They had a moderate ASD, mild septal flattening, and moderate RV dilation on echo. Similar to Case #6, this patient was also treated with pulmonary vasodilators and diuretics, with persistent signs of PH by echocardiogram which prompted hemodynamic testing. At baseline, PVRi was mildly elevated to 3.3 Wu*m2 and Qp:Qs was just 1.2:1. With the addition of 100% FiO2, mPAP remained stable (25 mmHg), PVRi improved to 2.12 Wu*m2, and Qp:Qs increased to 1.7:1. Given the improvement in PVRi and increase in relative pulmonary blood flow with vasodilation, transcatheter ASD closure was then successfully completed.

Despite a failed ASD closure in Case #5, there was modest improvement in echocardiographic markers of PAH at the follow-up echo with the addition of diuretics and discontinuation of iNO. While they demonstrated clinical improvement in their cardiopulmonary mechanics after this time, they ultimately were found not to be a surgical candidate due to their concurrent progressive liver failure (due to biliary atresia) and passed away after a prolonged hospitalization. Cases #6 and #7 both had significant improvement in septal flattening and RV dilation after shunt closures with eventual weaning of respiratory support from NIPPV to low flow nasal cannula (LFNC) and successful discharge home.

### Group 3: An infant with a multilevel shunt (case #8)

Case #8 highlights a previously healthy 9-month-old ex-term infant, who presented with acute respiratory distress secondary to an acute viral illness. Initial echocardiogram within 24 h of presentation showed moderate septal flattening, low-velocity left-to-right flow across a moderate-sized peri-membranous VSD, normal left heart size, moderate right ventricular enlargement and dysfunction, and suspicion of an unroofed coronary sinus (subsequently confirmed by computed tomogram), after which she was started on sildenafil and diuretic therapy. She had no other significant extracardiac anomalies. A subsequent echocardiogram after reaching therapeutic doses of sildenafil showed normalization of RV function with no significant changes in other echo findings. After resolution of her viral illness, a diagnostic catheterization was performed on hospital day 10. Invasive hemodynamics revelated an elevated mPAP of 35 mmHg, a mildly elevated PVRi of 3 Wu*m2 and an elevated Qp:Qs of 2.3:1 while on 25% FiO2. With these findings, she underwent surgical closure of both shunts on hospital day 12. Post-intervention, there was significant improvement in PH by echocardiography with only mild septal flattening and no RV dilation. In the post-operative period, the patient was maintained on sildenafil and able to wean from LFNC to room air with no evidence of acute cardiovascular or respiratory compromise.

## Discussion

Accurate understanding of hemodynamics in infants with significant prematurity or complex medical conditions is a critical component of their clinical care. However, this case series demonstrates how intracardiac shunts can substantially complicate this assessment when undertaken using echocardiography alone.

Echocardiography provides reasonably accurate estimates of pressure; however, non-invasive imaging remains limited in its ability to distinguish more nuanced physiological states in infants with concurrent intracardiac shunts and comorbid lung disease compared to isolated PAH. These states often exist along a continuum, from large shunts with high pressures and low pulmonary vascular resistance to minimal shunting with elevated pressures and increased pulmonary vascular resistance. As a result, determining the true hemodynamic profile of these high-risk infants is often challenging.

We present eight cases in which echocardiography findings were similar, suggesting elevated right-sided pressures in infants with a mix of pre- and post-tricuspid shunts. However, subsequent cardiac catheterization revealed two distinct hemodynamic profiles: either moderate shunts with modestly elevated PVRi (Cases #2, 4, 7, 8) or large shunts with elevated PVRi (Cases #1 and 6) responsive to vasodilatory testing. In most cases, these findings guided shunt closure or significant adjustments in medical therapy. Follow-up echocardiography demonstrated improvement in markers of pulmonary hypertension, RV size, and systolic function. Due to the small sample size and heterogeneity of this cohort, the impact of shunt closure on clinical trajectory or length of stay remains difficult to determine. At discharge, most patients continued pulmonary vasodilator therapy and required varying levels of respiratory support. Only one patient—the sole case without shunt closure—died during the study period from non-cardiac causes.

The clinical and echocardiographic profiles of these infants underscore that both pre-tricuspid (ASDs) and post-tricuspid (VSDs and PDAs) shunts can act as confounding factors in the echocardiographic assessment of pulmonary hypertension. Because the hemodynamic significance of a shunt depends not only on defect size but also on lesion type, “large” and “small” shunts were defined based on their overall physiological impact and catheterization findings rather than a uniform anatomical size threshold, recognizing the differing pulmonary vascular consequences of lesions such as ASDs and VSDs.

This phenomenon is well recognized for post-tricuspid shunts, which, depending on size, may equalize pressures between the right and left heart while imposing significant pressure and volume load on the pulmonary vasculature (21). Moreover, the patient's entire clinical status should be considered when interpreting their echocardiogram. As demonstrated in case #8, acute respiratory infection in the setting of a viral illness may also confound interpretation of a patient's hemodynamic state. In contrast, ASDs have traditionally been considered to primarily volume load the right heart without contributing to early pulmonary vascular remodeling (22). However, recent studies have hypothesized that infants with comorbidities that impact pulmonary vascular development such as BPD or CDH, this excessive volume load may further impair lung parenchymal and vascular growth (23–26).

The four infants in this series who underwent ASD closure (Cases #1, 3, 6, and 7) illustrate that volume loading from pre-tricuspid shunts can be associated with elevated right-sided pressures, although future work is needed to determine whether the relationship is causal or simply an association. These observations align with emerging evidence suggesting consideration of earlier ASD closure in the neonatal population than previously recommended (27–31). All four infants who underwent ASD closure in this series demonstrated improvement in echocardiographic markers of pulmonary hypertension, and three showed better respiratory status post-procedure (Cases #3, 6, and 7). However, given the small sample size we cannot definitively conclude that ASD closure alone was responsible for these changes, as the expected trajectory for infants with BPD is to have improved respiratory status with ongoing alveolar development.

Cases #1 and 6 also highlight how ASD closure can facilitate more aggressive pulmonary vasodilator therapy: both had baseline hemodynamics characterized by only modestly elevated Qp:Qs and elevated PVRi, both of which improved significantly with vasodilatory testing. It is worth noting that the increasing success of transcatheter defect closure in small infants in recent years has fundamentally changed the calculus regarding which infants are referred for intervention, given its markedly different risk profile compared with surgical repair. In some patients, eliminating the atrial-level shunt may allow for more effective intervention on the underlying pulmonary vascular disease, supporting management of both the excessive Qp and high PVR derangements in patients with mixed physiology. However, should device closure prove unsuccessful or anatomically unsuitable, a multidisciplinary discussion with close family involvement would be necessary to balance the risks of cardiopulmonary bypass and surgical defect closure against the anticipated clinical benefits.

## Limitations

This study was inherently limited by selection bias as these patients were purposefully chosen to be illustrative of a specific challenging clinical scenario where the echocardiographic and catheterization data did not clearly align. This report does not include the larger population of infants for whom either echo and catheterization data generally suggested similar physiology, or for whom catheterization was not performed. These are also findings from a single center at high altitude (∼1,600 m above sea level); thus, the clinical findings and practice patterns may not be applicable to all centers. As this was a retrospective study, more granular patient data such as respiratory rate, weight gain, and feeding tolerance was incomplete for some cases but would be important data to collect in future prospective trials, especially if we are to draw any conclusions about how shunt closure impacts a patient's clinical status. Moreover, the limited number of cases made statistical calculations impractical.

## Conclusions

Accurate assessment of cardiac hemodynamics in infants with complex medical conditions remains a critical yet challenging aspect of care. This case series highlights how cardiac shunts, both pre- and post-tricuspid, can significantly confound echocardiographic interpretation of pulmonary hypertension, often masking the true hemodynamic state. Invasive catheterization revealed distinct profiles not clearly delineated by echocardiography that guided targeted interventions, underscoring the limitations of non-invasive imaging in these scenarios. Affected infants may fit into one of three broad groups: former preterm infants with BPD, term infants with chronic respiratory failure secondary to CDH or genetic syndromes, and those with multi-level shunts. For these high-risk populations, judicious use of invasive hemodynamic testing should be considered either prior to initiation of pulmonary vasodilator therapy, or when echocardiographic findings persist despite standard therapy, as accurate characterization of physiology is essential for optimizing management and improving outcomes.

## Data Availability

The original contributions presented in the study are included in the article/Supplementary Material, further inquiries can be directed to the corresponding author/s.
